# USP18 Deficiency Presenting as Severe Neonatal Encephalopathy: A Case Report and Review of Pseudo-TORCH Syndromes

**DOI:** 10.7759/cureus.91952

**Published:** 2025-09-10

**Authors:** Amjed Alattas, Malak Alsamnan

**Affiliations:** 1 Neonatology, Prince Sultan Military Medical City, Riyadh, SAU

**Keywords:** interferonopathy, intracranial calcifications, neonatal coma, neonatal encephalopathy, pseudo-torch, usp18 deficiency

## Abstract

Pseudo-TORCH (Toxoplasma, “Other” agents, Rubella, Cytomegalovirus, Herpes simplex, etc.) syndromes are rare genetic disorders that mimic congenital TORCH infections but lack an infectious cause. Affected infants present with encephalopathy, intracranial calcifications, and congenital anomalies. We report a term male infant who developed catastrophic encephalopathy and coagulopathy on day eight of life, with neuroimaging showing hemorrhages, calcifications, and cortical malformations. Whole-exome sequencing revealed a homozygous splice-site mutation in USP18, confirming pseudo-TORCH syndrome type 2. Despite maximal intensive care, the infant succumbed at one month of age. This case illustrates the diagnostic challenges and rapid progression of USP18 deficiency, highlights the value of early genetic confirmation for family counseling, and reviews the spectrum of pseudo-TORCH syndromes. Emerging therapies targeting interferon pathways may offer future hope. Our literature review highlights the differential diagnoses and genetic variability among pseudo-TORCH conditions, which include mutations in OCLN, USP18, STAT2, and JAM3. This emphasizes the importance of early genetic confirmation for better prognosis and family counseling. Emerging therapies targeting the interferon pathway, such as Janus kinase (JAK) inhibitors, are currently under investigation and may offer hope for these previously untreatable disorders. This case underscores the necessity of considering pseudo-TORCH syndromes in neonates who present with unexplained encephalopathy and brain calcifications after infectious causes have been ruled out.

## Introduction

Congenital infections classified under the TORCH acronym (Toxoplasma, “Other” agents, Rubella, Cytomegalovirus, Herpes simplex, etc.) typically cause a triad of neonatal signs: microcephaly, intracranial calcifications, and neurodevelopmental impairment, often with systemic features, such as rash, hepatosplenomegaly, and jaundice. However, not all infants with this presentation have an infection. The term “pseudo-TORCH” was first introduced in the early 1980s after Baraitser et al. described two brothers with severe congenital microcephaly and extensive brain calcifications born to infection-negative parents [1]. In 1994, Reardon et al. reported an autosomal recessive “intrauterine infection-like” syndrome in multiple children from a consanguineous family (now known as pseudo-TORCH syndrome type 1 or Baraitser-Reardon syndrome) [2]. These cases established that a TORCH-like clinical picture could result from an inherited condition rather than an infection. It is now recognized that pseudo-TORCH syndromes are genetic interferonopathies, disorders caused by mutations leading to abnormal upregulation of type I interferon signaling and an “in utero cytokine storm” that damages the developing brain [3,4]. In recent years, multiple genetic causes of pseudo-TORCH phenotypes have been identified in the literature. To date, three main subtypes have been defined (all autosomal recessive), each caused by mutations in a different gene involved in neurodevelopment or in interferon regulation. We present a case of pseudo-TORCH syndrome type 2 (USP18 deficiency) in a term neonate and provide a comprehensive literature review of pseudo-TORCH syndromes, highlighting their distinguishing features, differential diagnoses (notably Aicardi-Goutières syndrome), and emerging treatments.

## Case presentation

Patient and initial presentation

The patient was a male newborn delivered at 38 weeks’ gestation to a healthy mother (gravida 3, para 2) via a spontaneous vaginal delivery. Birth weight was 2,140 g (below the 3rd percentile), length was 45 cm (3rd percentile), and head circumference was 32 cm (18th percentile). The parents were first cousins with no family history of similar illnesses or neonatal deaths. Aside from asymmetrical intrauterine growth restriction, the only notable finding at birth was a 1.8 × 1.0 cm soft, mobile mass on the left lateral neck with a necrotic center, which was surgically excised on the first day of life. The infant’s Apgar scores were normal, and there were no dysmorphic features. He was admitted to the intermediate care nursery for routine observation and evaluation of the neck mass. Ultrasound of the neck revealed an ill-defined heterogeneous area with increased echogenicity (possibly calcification or fat) anteromedially to the left sternocleidomastoid muscle. This appearance is inconsistent with cervical neuroblastoma or branchial cleft anomalies. The location is also unusual for midline fusion defects or dermoid cysts. Over the first week of life, the infant tolerated enteral feeds, the neck lesion regressed post-excision, and he remained stable.

Neurologic deterioration

On day eight of life, the infant developed poor feeding, lethargy, and hypotonia, which rapidly progressed to unresponsiveness (coma) with decerebrate posturing. The patient exhibited intermittent tonic extensor seizures. The infant was intubated for airway protection and transferred to the neonatal intensive care unit (NICU).

Initial workup

Given the sudden onset of neonatal encephalopathy, an infectious process was first suspected. Empiric broad-spectrum antibiotics were initiated, and a comprehensive evaluation for sepsis was performed. Blood, urine, and cerebrospinal fluid cultures were sterile. All urgent TORCH infection studies were negative, including tests for cytomegalovirus (urine PCR), toxoplasmosis (IgM/IgG), rubella (IgM), herpes simplex virus (CSF PCR), syphilis (RPR), and Zika virus (PCR). Metabolic screening (plasma amino acids, urine organic acids, ammonia, and blood glucose) revealed no evidence of inborn errors of metabolism. The liver function tests were within normal limits. Complete blood count (CBC) and coagulation profile are summarized in Table 1.

**Table 1:** Summary of the complete blood count and coagulation results. The arrow denotes a drop from the initial to the subsequent hemoglobin value within 24 hours. WBC: white blood cell count; Hgb: hemoglobin; Plt: platelet count; INR: international normalized ratio; PT: prothrombin time; aPTT: activated partial thromboplastin time

Parameter	Result (Patient)	Reference Range (units)
WBC	10.5 × 10^9^/L	5–21 × 10^9^/L
Hemoglobin (Hgb)	16.3 → 12.3 g/dL*	13.5–21 g/dL
Platelets (Plt)	74 × 10^9^/L	150–450 × 10^9^/L
INR	2	0.9–1.3
PT	25.9 s	11.5–14.5 s
aPTT	79 s	30–41 s

Neuroimaging findings

**Figure 1:**
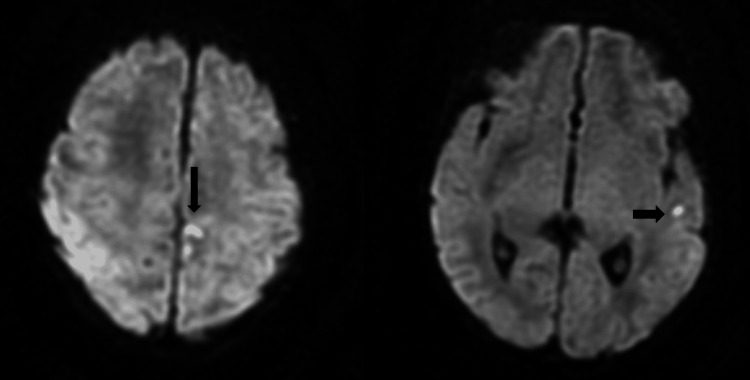
MRI brain diffusion-weighted imaging (DWI). MRI brain showed multiple areas of high DWI intensities, with associated low ADC signal intensities involving the bilateral parietal parasagittal region and right cortex (black arrows).

Emergent cranial ultrasound and brain MRI (day three of illness) revealed extensive intracranial abnormalities. There were multiple acute intraparenchymal echogenic foci. Echogenic lesions were also present in the cerebellum and subarachnoid space (Figure 1). In addition, widespread punctate calcifications were visible on T1-weighted MRI (confirmed as echogenic foci on cranial ultrasound) distributed in a band-like pattern along the cerebral cortex, basal ganglia, and thalami. The cortex appeared abnormally simplified, with regions suggestive of polymicrogyria. This combination of diffuse brain calcifications, cortical malformations, and multifocal hemorrhages strongly suggests a prenatal insult leading to “TORCH-like” brain injury. Notably, all infectious disease tests were negative.

Supportive treatment

The infant was managed with maximal supportive therapy in the NICU. Mechanical ventilation was required due to apnea and coma; at times, high-frequency oscillatory ventilation and inotropic support were necessary to treat refractory hypotension and pulmonary hemorrhage. Coagulopathy was managed with fresh frozen plasma, cryoprecipitate, and vitamin K administration. Phenobarbital was initiated for seizure control. Given the combination of intracranial calcifications and a negative infectious workup, high-dose corticosteroids were empirically administered to address a possible neuroinflammatory etiology. Despite these interventions, the neonate remained comatose with absent brainstem reflexes (fixed pupils and no spontaneous respirations) by the end of the first week of life, indicating a poor prognosis.

Genetic testing and diagnosis

Given the negative infectious and metabolic test results and the clinical suspicion of pseudo-TORCH syndrome, we pursued genetic evaluation. Rapid whole-exome sequencing (WES) was performed on the infant and both parents. WES identified a homozygous variant in the USP18 gene in the patient: a single-nucleotide substitution at the splice-donor site of intron 10 (NM_017414.3: c.1073+1G>A). This pathogenic variant is predicted to cause aberrant splicing and loss of USP18 function. Notably, the same mutation has been reported in multiple patients with pseudo-TORCH syndrome type 2. Both parents were confirmed to be heterozygous carriers, consistent with an autosomal recessive inheritance pattern. No other significant genetic findings were identified to explain the infant’s condition. The genetic diagnosis was therefore pseudo-TORCH syndrome type 2, also known as USP18 deficiency.

Hospital course and outcome

Confirmation of USP18 deficiency allowed us to counsel the family regarding the severe prognosis associated with complete USP18 loss-of-function. Currently, no established treatment or cure is available for this condition. Experimental therapy with a Janus kinase (JAK) inhibitor was considered based on recent reports of interferon-blocking treatments used in similar interferon-mediated disorders. However, given the patient’s critical condition (deep coma and ventilator dependence with multiorgan failure), he was not considered a candidate for aggressive experimental intervention. Care was refocused on comfort measures. The infant died at approximately one month of age from refractory hypotensive shock and multiorgan failure. Congenital cervical lymphatic malformation was judged to be an unrelated coexisting condition and did not impact the clinical course.

## Discussion

Our patient exemplifies the catastrophic course of pseudo-TORCH syndrome type 2 (USP18 deficiency). He was initially stable during the first week of life but experienced sudden and severe deterioration on day eight, progressing rapidly to seizures, coagulopathy, coma, and multiorgan failure. Neuroimaging revealed widespread calcifications, hemorrhages, and cortical malformations, a pattern highly suggestive of an in utero inflammatory insult. Hematologic abnormalities, including thrombocytopenia and consumptive coagulopathy, likely reflected systemic inflammatory activation, while pulmonary hemorrhage and refractory shock further illustrated the multisystem hyperinflammatory state. Despite maximal intensive care - including ventilation, inotropic support, blood product replacement, and empiric corticosteroids - the infant’s condition continued to worsen, and he ultimately died at one month of age. Whole-exome sequencing later confirmed USP18 deficiency, but the diagnosis became available only after his fatal decline, underscoring the challenge of relying on genetic confirmation in rapidly progressive neonatal disease.

Pseudo-TORCH syndrome overview

Pseudo-TORCH syndrome refers to a group of inherited conditions that clinically and radiologically resemble congenital infections (TORCH) but are not caused by any infectious agent. Pseudo-TORCH syndromes are now known to result from genetic mutations that lead to pathological upregulation of type I interferon activity during fetal development. The excessive interferon signaling causes a severe inflammatory response that injures the developing brain in utero - essentially a cytokine-mediated “storm” that mimics the pattern of damage seen in congenital infections [3,4]. Over the past decades, research has shown that pseudo-TORCH presentations are genetically heterogeneous. There are three primary subtypes defined so far, all autosomal recessive, each linked to mutations in a different gene. In addition, at least one other rare genetic disorder (involving JAM3) produces a similar “pseudo-TORCH” pattern. Below, we summarize the main subtypes and related conditions, highlighting distinguishing features and molecular causes.

Pseudo-TORCH Syndrome Type 1 (PTORCH1)

This subtype (also called Baraitser-Reardon syndrome) is caused by biallelic mutations in OCLN, which encodes occludin, a tight-junction protein critical for blood-brain barrier integrity. Neuroimaging reveals a characteristic band-like calcification with simplified gyration and polymicrogyria (BLC-PMG) pattern, characterized by bilateral bands of calcification at the cortical-subcortical junction, accompanied by markedly simplified cortical folding and areas of polymicrogyria on brain MRI [5,6]. Clinically, PTORCH1 is typically diagnosed at birth with microcephaly, widespread intracranial calcifications (often exhibiting a band-like distribution), polymicrogyria, and early-onset intractable seizures [7,8]. Systemic features such as hepatic dysfunction or thrombocytopenia may occur, but are less consistent than the profound neurological impairment. OCLN mutations were first identified as the cause of pseudo-TORCH 1 in 2010 by O’Driscoll et al. [8], and numerous cases have since been reported. Recent case reports have expanded the phenotype beyond the brain; for instance, Ekinci et al. (2020) described a child with a homozygous OCLN frameshift mutation who developed central diabetes insipidus and renal salt-wasting in infancy, in addition to the classic neurological features of PTORCH1 [9]. This suggests that the role of occludin may extend to other organ systems (e.g., the hypothalamic-pituitary axis and kidneys), although severe cerebral malformations and neurological disability remain the defining features.

Pseudo-TORCH Syndrome Type 2 (PTORCH2)

This subtype is caused by biallelic mutations in USP18, which encodes ubiquitin-specific peptidase 18 - a key negative regulator of type I interferon signaling. USP18 mutations were first identified in 2016 by Meuwissen et al. in infants with severe TORCH-like neonatal encephalopathy [4]. Under normal conditions, USP18 acts as a “gatekeeper” that turns off interferon-α/β signaling by binding the IFNAR2 receptor subunit, thereby preventing excessive JAK1-STAT activation. Loss of USP18 function leads to unrestrained interferon-driven inflammation, effectively triggering a cytokine storm in the developing fetus. Clinically, PTORCH2 is one of the most severe forms of pseudo-TORCH syndrome. It often presents in utero with diffuse intracranial calcifications and brain destruction visible on prenatal imaging and can even cause antenatal intracerebral hemorrhages. Newborns with USP18 deficiency frequently experience profound neurological depression (often progressing to coma in the neonatal period), refractory seizures, and signs of systemic inflammation such as markedly elevated inflammatory markers, hepatosplenomegaly, and even skin necrosis due to vasculopathy [4]. Our patient’s rapid decline into coma, seizures, coagulopathy, and diffuse brain hemorrhage is consistent with other reported PTORCH2 cases. Multiorgan involvement is common, including severe coagulopathy, acute respiratory failure, and shock reflecting a hyperinflammatory state affecting multiple systems. The mortality rate in complete USP18 deficiency has historically been extremely high; nearly all reported patients with biallelic null USP18 mutations have died in the neonatal period or utero [4,10]. PTORCH2 is considered one of the most severe genetic interferonopathies and is typically lethal unless the interferon response can be attenuated.

Pseudo-TORCH Syndrome Type 3 (PTORCH3)

This subtype is caused by homozygous gain-of-function mutations in signal transducer and activator of transcription 2 (STAT2). STAT2 is a critical signaling molecule immediately downstream of the type I interferon receptor, and its activity is usually kept in check by USP18. In 2019, Duncan et al. [10] identified a homozygous missense mutation in STAT2 (R148Q) in an infant with a severe interferonopathy; this mutation prevented STAT2 from being regulated adequately by USP18, thereby blocking the negative feedback control of interferon signaling. Gruber et al. [11] later reported another patient with a STAT2 gain-of-function mutation, which confirmed this disease mechanism. Clinically, PTORCH3 closely resembles PTORCH2: affected neonates present with severe encephalopathy, often evident from birth or early infancy, accompanied by microcephaly, diffuse intracranial calcifications, refractory seizures, and systemic inflammatory features. Some STAT2 gain-of-function cases have exhibited a relapsing or episodic course - for example, episodes of high fever, cytokine surges, and worsening inflammation triggered by infections or other stressors. This relapsing-remitting pattern may reflect that specific STAT2 mutations cause a slightly lower degree of interferon hyperactivity compared to complete USP18 loss, allowing intermittent partial recovery [12].

JAM3-related syndrome (HDBSCC)

In addition to the three defined pseudo-TORCH subtypes above, other rare genetic disorders can produce a “pseudo-TORCH” pattern. One example is the syndrome of hemorrhagic destruction of the brain, subependymal calcification, and congenital cataracts (HDBSCC) caused by mutations in JAM3 (which encodes junctional adhesion molecule 3). De Rose et al. recently reported an Italian neonate with a novel homozygous JAM3 variant who presented with this syndrome [13]. The HDBSCC disorder, first described by Mochida et al. in 2010 [14], is characterized by prenatal-onset catastrophic brain injury. Affected infants often show evidence of widespread intracerebral hemorrhages already in utero or at birth, along with subependymal (periventricular) calcifications and congenital cataracts. This triad of intracerebral hemorrhages, calcifications, and cataracts is pathognomonic for JAM3-related disease.

Diagnostic workup and differential diagnosis

Diagnosing a suspected pseudo-TORCH syndrome in a neonate requires both excluding congenital infections and pursuing targeted genetic testing. In any critically ill newborn with unexplained intracranial calcifications and encephalopathy, the first step is to thoroughly rule out intrauterine infections (TORCH) using appropriate cultures, PCR assays, and serologies. In our case, the comprehensive infectious evaluation was completely negative, which directed attention to an inherited etiology. Neuroimaging proved to be a crucial clue: the combination of intracranial calcifications, migrational brain abnormalities (e.g., polymicrogyria), and early neonatal hemorrhagic lesions strongly suggested a prenatal insult. It was highly typical of a pseudo-TORCH syndrome. Once infections are excluded, rapid genetic testing - via whole-exome sequencing or a targeted gene panel (including at least OCLN, USP18, and STAT2) - should be performed to confirm the diagnosis. The differential diagnosis overlaps with that of other interferonopathies, particularly Aicardi-Goutières syndrome (AGS). AGS typically manifests later in infancy, characterized by subacute regression, cerebrospinal fluid lymphocytosis, elevated interferon-α levels, and chilblain skin lesions [3,15,16]. In contrast, pseudo-TORCH infants often present at or immediately after birth with severe encephalopathy, coagulopathy, and systemic inflammation.

Management and prognosis

Currently, there is no cure for pseudo-TORCH syndromes, and prognosis remains poor. Management is primarily supportive and symptom-based. Affected neonates typically require prolonged intensive care, including mechanical ventilation for respiratory failure or apnea, inotropic support for shock, and anticonvulsant therapy for seizures. Nutritional support is often necessary via tube feeding, and vigilant monitoring for complications such as hydrocephalus, spasticity, and coagulopathy is essential. Our patient required blood product transfusions, including plasma and cryoprecipitate, for consumptive coagulopathy, reflecting the systemic inflammatory cascade typical of USP18 deficiency.

A multidisciplinary team, including neonatologists, neurologists, geneticists, immunologists, hematologists, and surgeons, is essential for comprehensive care. In our case, surgical evaluation was required for a large cervical lymphangioma, illustrating the importance of addressing coexisting conditions independently. Once a genetic diagnosis is achieved, counseling is critical, especially in consanguineous families where recurrence risk is high.

Emerging insights into the pathophysiology of PTORCH2 and PTORCH3 have prompted experimental use of therapies that target interferon signaling. JAK inhibitors such as ruxolitinib have been reported in isolated cases, with one Saudi infant with USP18 deficiency surviving beyond infancy after treatment [15], in contrast to the otherwise universally fatal course [4,10]. Monoclonal antibodies targeting interferon-α are another potential avenue, though evidence remains anecdotal. These approaches remain experimental and, critically, depend on early diagnosis before irreversible brain injury occurs.

The extreme rarity of pseudo-TORCH syndromes makes controlled trials challenging. International registries and collaborative studies are urgently needed to define natural history, evaluate experimental therapies, and guide future interventions. Until then, meticulous and supportive care, along with early genetic testing, remains the cornerstone of management.

This report has several limitations. Functional validation of the identified USP18 splice-site variant (e.g., RNA studies) and immunological testing (including interferon signature analysis) were not performed, and pathological confirmation through autopsy was not available. Furthermore, the patient’s rapid deterioration and death occurred before whole-exome sequencing results were returned, precluding additional investigations or the consideration of experimental therapies. These limitations reflect the real-world challenges of diagnosing and managing ultra-rare, fulminant neonatal disorders; however, they are important to acknowledge for transparency and scientific rigor.

## Conclusions

This case of USP18 deficiency demonstrates the devastating neonatal course of pseudo-TORCH syndromes and reinforces their inclusion in the differential diagnosis of encephalopathy, intracranial calcifications, and negative infectious evaluations in infants. Early neuroimaging combined with rapid genetic testing is essential for timely diagnosis, accurate counseling, and effective family planning. Although the prognosis remains poor, reports of interferon-targeted therapies, such as JAK inhibitors, highlight a potential window for intervention if identified early. Until such therapies are validated, multidisciplinary supportive care remains the cornerstone of treatment. Continued reporting of new cases and collaborative registry efforts are crucial for improving recognition, expanding therapeutic experience, and guiding future treatment strategies for these rare disorders.
